# An efficient eco advanced oxidation process for phenol mineralization using a 2D/3D nanocomposite photocatalyst and visible light irradiations

**DOI:** 10.1038/s41598-017-09826-6

**Published:** 2017-08-29

**Authors:** H. Al-Kandari, A. M. Abdullah, Yahia H. Ahmad, S. Al-Kandari, Siham Y. AlQaradawi, A. M. Mohamed

**Affiliations:** 1 Department of Health Environment, College of Health Sciences, Public Authority for Applied Education and Training, P.O. Box 1428, Faiha 72853, Kuwait; 2 Center for Advanced Materials, Qatar University, Doha, P.O. Box 2713 Qatar; 3 Chemistry Department, College of Arts and Sciences, Qatar University, Doha, P.O. Box 2713 Qatar; 4 Department of Chemistry, Faculty of Science, Kuwait University, P.O. Box 5969, Safat, 13060 Kuwait

## Abstract

Nanocomposites (CNTi) with different mass ratios of carbon nitride (C_3_N_4_) and TiO_2_ nanoparticles were prepared hydrothermally. Different characterization techniques were used including X-ray diffraction (XRD), UV-Vis diffuse reflectance spectroscopy (DRS), X-ray photoelectron spectroscopy (XPS), transmission electron spectroscopy (TEM) and Brunauer-Emmett-Teller (BET). UV-Vis DRS demonstrated that the CNTi nanocomposites exhibited absorption in the visible light range. A sun light - simulated photoexcitation source was used to study the kinetics of phenol degradation and its intermediates in presence of the as-prepared nanocomposite photocatalysts. These results were compared with studies when TiO_2_ nanoparticles were used in the presence and absence of H_2_O_2_ and/or O_3_. The photodegradation of phenol was evaluated spectrophotometrically and using the total organic carbon (TOC) measurements. It was observed that the photocatalytic activity of the CNTi nanocomposites was significantly higher than that of TiO_2_ nanoparticles. Additionally, spectrophotometry and TOC analyses confirmed that degraded phenol was completely mineralized to CO_2_ and H_2_O with the use of CNTi nanocomposites, which was not the case for TiO_2_ where several intermediates were formed. Furthermore, when H_2_O_2_ and O_3_ were simultaneously present, the 0.1% g-C_3_N_4_/TiO_2_ nanocomposite showed the highest phenol degradation rate and the degradation percentage was greater than 91.4% within 30 min.

## Introduction

Semiconductor photocatalysis is considered an alternative and environmentally benign technology in the field of wastewater treatment and for renewable energy sources. Although many semiconductors were tested as photocatalysts, TiO_2_ was intensively examined due to its nontoxicity, photostability, availability and low cost^1–6^. Nevertheless, there are several barriers concerning the use of TiO_2_ on a large scale. For example, TiO_2_ has a relatively high band gap energy (3.35 eV). This high band gap energy allowed the use of only UV radiation in photocatalysis, which is only 4% of the total solar energy^7, 8^. In addition, the high electron-hole recombination rate during the photocatalysis of TiO_2_ affects its activity. Therefore, numerous ideas and studies were developed to enhance its photocatalytic performance. These studies included changing the experimental conditions and synthesis methods of TiO_2_^9–13^, metal particle loading^5, 6, 14^ and using a co-catalyst^5, 6, 15^. Considerable attention has been focused on utilizing carbonaceous nanomaterials as a support for TiO_2_ to improve its photocatalytic performance due to their unique electrical properties and controllable structures^7, 16–23^. An example of these supports is graphitic carbon nitride (g-C_3_N_4_), which can be obtained from the pyrolysis of nitrogen-rich organic precursors and is considered the most stable allotrope of carbon nitride^14^. Recently, Ong *et al*. reviewed numerous studies regarding g-C_3_N_4_-based photocatalysis^24^. Additionally, several researchers have reported that coupling g-C_3_N_4_ with TiO_2_ may improve the photocatalytic behavior of TiO_2_ by lowering its band gap energy and slowing the electron-hole recombination rate^5, 14, 16, 25, 26^.

Phenol, among numerous organic pollutants found in wastewater, requires great attention due to its toxicity^27, 28^. Its existence has been confirmed in several industrial wastewaters, including chemical and petrochemical industries. Phenol treatment is expensive since it is unresponsive to traditional treatment techniques^1, 29^. Therefore, current investigations are directed at advanced oxidation processes (AOP) as a replacement for traditional techniques.

Although a few research studies targeted the use of g-C_3_N_4_/TiO_2_ composites in photocatalysis, most investigations were directed towards the degradation of dyes in the absence of oxidizing agents and using UV irradiation, which significantly increases the cost^5, 6, 9–11, 14, 15, 17, 26, 30–36^. In this work, the focus is on investigating the extremely enhanced kinetics of the advanced oxidation process in which the photocatalytic treatment of a high concentration of phenol (20 ppm), which is more difficult to mineralize than dyes, was done using a hydrothermally-prepared nanocomposite of 0.1% g-C_3_N_4_ nanosheets on top of TiO_2_ nanoparticles and a low-power Xe irradiation lamp (mainly irradiates visible light) as a simulator for sunlight. Furthermore, the effect of the *in-situ* addition of eco oxidants e.g. hydrogen peroxide (H_2_O_2_) and/or ozone (O_3_) (generated using a home-made electrode) on the rate of photocatalytic degradation of phenol was also included and, to our knowledge, this work has not been previously addressed.

## Experimental

### Synthesis

All chemicals were of analytical grade and no extra purification treatments were carried out. Graphitic carbon nitride (g-C_3_N_4_) was synthesized, as reported by Wang *et al*.^37^. Specifically, melamine (Alfa Aesar, 99% pure) was placed in a covered crucible and heated under stationary air conditions at 550 °C with a ramping rate of 2.5 ° min for 4 h. The resulting carbon nitride powder, which was pale yellow in color, was washed with deionized water and then dried under vacuum at 60 °C.

The g-C_3_N_4_–TiO_2_ nanocomposite (CNTi) photocatalyst was prepared by loading various mass ratios of CN on a commercial P25 TiO_2_ (Sigma Aldrich, 21 nm particle size) support as follows. TiO_2_ was added to an absolute ethanol/deionized water mixture at a 1:1 ratio and sonicated for 30 min. The same exact procedure was conducted with CN. Afterwards, the two aforementioned suspensions were mixed together, further sonicated for 30 min and nitric acid/ ammonium hydroxide solutions were used to adjust the pH to 3.5. The resultant mixture was poured into a Teflon-lined stainless steel autoclave and left overnight at 120 °C. The suspension was centrifuged, later washed with hydrochloric acid (1 M) and then with deionized water. Lastly, the product was dried overnight at 80 °C. The loading percentages of C_3_N_4_ on TiO_2_ were designed to be 0.1, 0.5 and 1.0% and the corresponding names are 0.1CNTi, 0.5CNTi and 1CNTi, respectively.

### Characterization

X-ray diffraction (XRD) measurements were conducted using a Bruker D8 diffractometer equipped with a Lynxeye detector and a Cu K_α_ radiation source (λ  = 15.406 × 10^−2^ nm). The diffractometer operated at 40 mA and 40 kV. The scanning was performed from 10–80° using a scan step of 0.015° and a step time of 0.2 s. To determine the different phases, an automatic JCPDS library search and match was used. Additionally, standard SERACH and DIFFRACT AT (Australia) computer software packages were employed.

Raman spectra were obtained using an InVia Raman microspectrometer, which worked under macro conditions (*f* = 3 cm) with a 785 nm excitation line and a laser control of approximately 2 mW. The measurements were conducted without prior treatment and average spectra were recorded from five registration scans. The spectra ranged from 100–3500 cm^−1^ with three accumulation numbers and a 10 s exposure time for each registration.

UV-Vis diffuse reflectance spectroscopy (DRS) was performed using a Cary 5000 UV-Vis-NIR (Agilent, Australia) equipped with an integrating sphere accessory. The test samples were supported by KBr, and the measured spectra were recorded at room temperature from 200 to 800 nm with a resolution of 0.05 nm.

The XPS spectra were obtained using Thermo Scientific ESCALAB-250Xi spectrometer. A monochromatic AlK_α_ radiation source that operated at a power of 300 W (20 mA, 15 kV), was utilized. The vacuum in the analysis chamber was less than 7 × 10^−9^ Torr during the measurements. Carbon pollution that was referenced to C1s at 284.8 eV with a ±0.2 eV experimental error was taken as a base for elemental binding energies.

The amount of carbon, hydrogen and nitrogen were measured using a GmBH analyzer CHNS-O EA.

The BET (Brunauer-Emmett-Teller) surface areas were determined by an automatic ASAP 2010 Micromeritics sorpometer (USA) that was equipped with an outgassing platform and an online acquiring data and handling system, which functioned using numerous computer-run methods to examine the adsorption data.

Thermogravimetric analyses (TGA) were achieved utilizing a Perkin Elmer, USA/Pyris 1 TGA instrument. The air flow was heated at 5 °C min^−1^ to quantify the exact loading percentage of the carbonaceous material (CN) on the TiO_2_ support.

The CNTi nanocomposite was characterized using a Japanese JEOL 2100 F high-resolution transmission electron microscopy (HR-TEM) operating at 200 kV and coupled with an energy dispersive x-ray unit. The sample preparation was conducted by mixing a small quantity of sample with ethanol and sonicating for 15 min. The sample was then loaded onto the TEM grid.

A SiO_2_/Ti (300 nm)/Pt (100 nm)/TiOx (100 nm)/(SnOx500 nm) electrode was used to generate ozone from a 0.5 M NaOH solution at 2 V versus the Ag/AgCl reference electrode. The electrode was manufactured at The Pennsylvania State University (USA) Materials Research Institute Nanofabrication Laboratory using a KJL CMS-18 sputtering system. The films were grown at room temperature on a quartz sample (SiO_2_) at 5 mtorr and 200 watts. The metallic films (Ti and Pt) were grown under an argon atmosphere, whereas the oxide films were grown in a mixture of 15% O_2_ in Ar. The generated ozone was transferred using Tygon tubing to the photocatalytic reactor. The electrode had an ozone production efficiency close to 20% (excluding the dissolved ozone, which was not measured in the generating solution).

### Photocatalytic experiments

For every photocatalytic run, 20 ppm of a phenol aqueous solution was prepared using deionized water. A 0.1 g catalyst was added to 100 mL of the phenol solution and stirred in the dark for 30 min until the adsorption equilibrium was achieved. The pH of the phenol solution was not adjusted. The photoreaction vessel was irradiated by a xenon lamp (150 W) without a cut-off filter at an integrated intensity of 12 mW cm^−2^ at 4.5 cm. The beginning of the experiment (time zero) occurred when the lamp was turned on. Samples were drawn every 5 min from the reaction vessel and filtered to dispose of the suspended catalyst particles through a nylon filter membrane with a 0.4 μm porosity. The UV-Vis spectrophotometer was used to determine the amount of phenol at a maximum absorbance of 298 nm. Experiments that involved O_3_, were electrochemically produced in an isolated cell that contained 0.5 M NaOH using a SiO_2_/Ti (300 nm)/Pt (100 nm)/TiOx (100 nm)/SnOx (500 nm) electrode with a maximum current of 3000 mA at 8 V. The undissolved portion of the generated ozone was transferred to the photoreactor using a Tygon tube. A 4 ppm quantity of electrochemically generated ozone gas was used for each photocatalytic run. The photocatalysis system that included an O_3_ generating unit (electrochemical cell plus a potentiostat), an ozone meter, a UV lamp chiller and the photocatalytic reactor is shown elsewhere^38^.

Finally, after photocatalytic experiments, the total organic carbon (TOC) for all phenol solutions were measured using a TOC-VPH analyzer, Shimadzu, Japan.

## Results and Discussion

### Characterization

**Figure 1:**
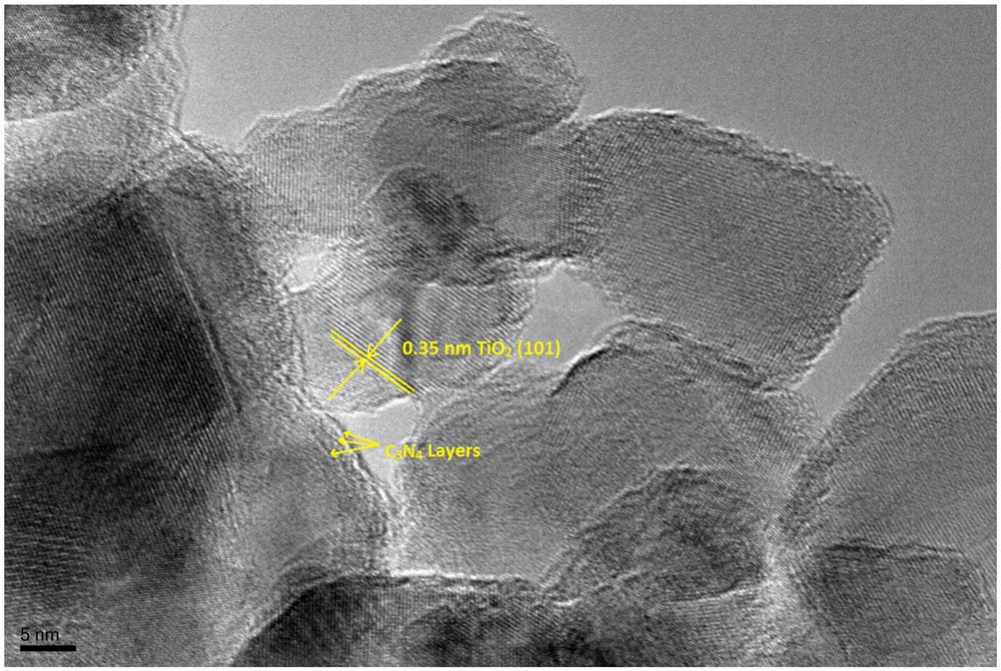
HR-TEM Micrograph of the 1CNTi composite.

Figure 1 displays a HR-TEM micrograph for the 1CNTi nanocomposite loaded on the TiO_2_ support. It is evident that the average particle size is approximately 20 nm and C_3_N_4_ was uniformly distributed on TiO_2_. It also shows the lattice spaces of TiO_2_ (0.35 nm) and the lattice fringes of C_3_N_4_.

**Figure 2:**
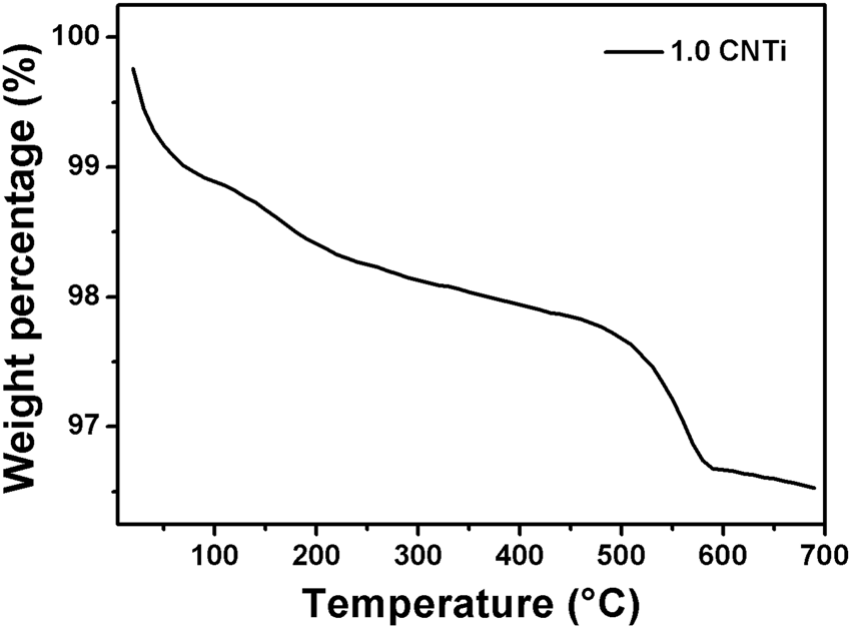
TGA of the 1CNTi composite.

To determine the C_3_N_4_ contents in the final products, TGA was employed from 20 to 700 °C in air. Pure TiO_2_ shows almost no weight loss in the studied temperature range. However, pure C_3_N_4_ experienced rapid weight loss from 550 to 640 °C, which indicates its decomposition. For the CNTi nanocomposite, the weight loss increased at temperatures above 500 °C. At the end of the analysis, the total combustion of CN was achieved. Therefore, the weight loss is related to the amount of CN in the composite. The results obtained from the TG studies indicated that the CN content in the respective composites corresponded to nominal values. For example, Fig. 2 shows the TGA of the 1CNTi composite.

**Figure 3:**
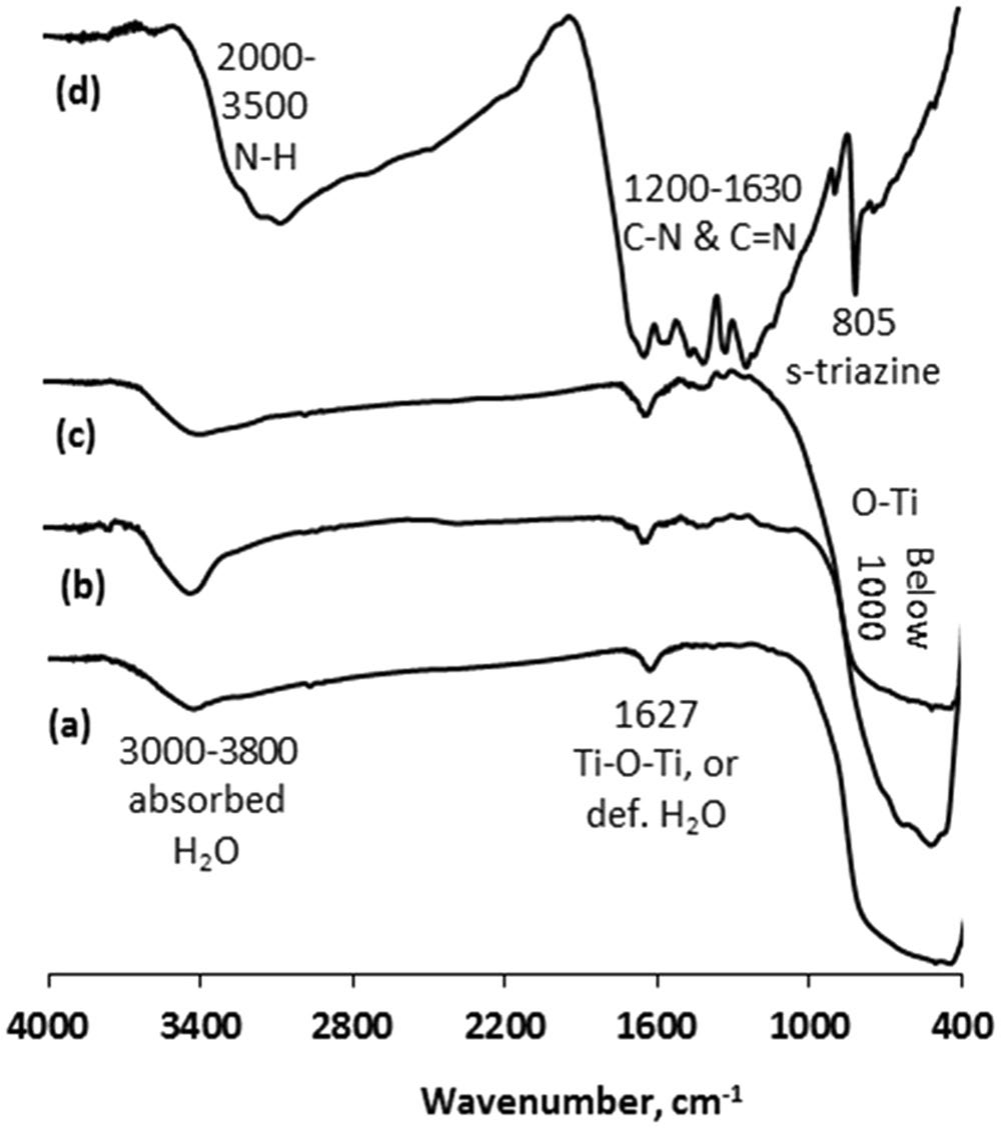
FT-IR spectra of (**a**) TiO_2_, (**b**) 0.1CNTi, (**c**) 1CNTi and (**d**) C_3_N_4_.

Figure 3 displays FT-IR spectra of TiO_2_, CNTi and C_3_N_4_. In Fig. 3a, a peak was detected at 1627 cm^−1^, which was ascribed to deformed water molecules or a Ti-O-Ti stretching vibration^22^. Additionally, the low frequency strong and broad absorption band (below 1000) was assigned to the Ti-O-Ti vibration in TiO_2_^39–42^. The broad band in the range of 3800 to 3000 cm^−1^ was designated as O-H stretching vibrations of C-OH groups (from carboxylic groups) or/and intercalated water molecules. The C_3_N_4_ spectrum in Fig. 3d showed several strong bands from 1130 cm^−1^ to 1639 cm^−1^, which were associated with stretching and rotation vibrations of C-N and C = N in heterocycles^8^. The band at 805 cm^−1^ was related to s-triazine ring vibrations and the wide absorption peak at 2000–3500 cm^−1^ was attributed to the N-H bond^8, 10, 11, 31, 43^. The main characteristic peaks of C_3_N_4_ were reduced and several peaks disappeared after loading on TiO_2_. These data indicated a low percentage of C_3_N_4_ in the composites (Fig. 3b,c).

**Figure 4:**
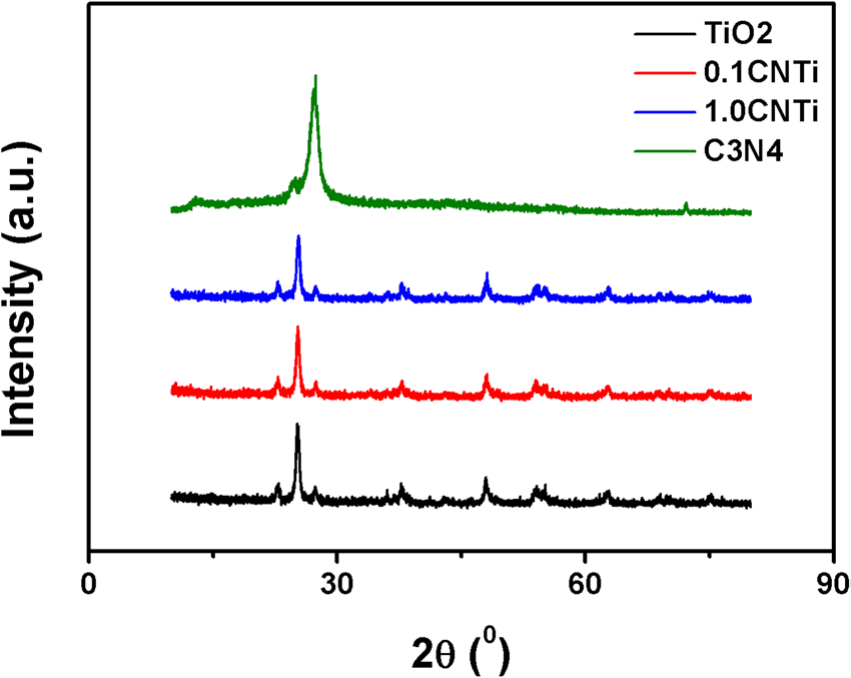
XRD patterns of (**a**) TiO_2_, (**b**) 0.1 CNTi, (**c**) 0.5 CNTi, and (**d**) 1.0 CNTi and (**e**) C_3_N_4_.

The XRD measurements for bare TiO_2_ (Fig. 4a) show diffraction patterns of both anatase and rutile phases. The peaks located at 25.4, 37.9, 48.0, 54.0, 55.1, 62.8, 69.1, 70.5 and 75.2° were assigned to the anatase phase (JPDS 21–1272), whereas the peaks assigned to the rutile phase were obtained at 27.5, 36.1 and 41.1° (JPDS 21–1276)^23, 44^. Figure 4e shows two distinctive diffraction peaks: a strong peak located 27.4° with an interlayer spacing of 3.2 Å and a broad peak located at 13.1° with an interlayer spacing of 6.8 Å, which can be indexed to the hexagonal phase of graphitic C_3_N_4_. The former peak was related to the (002) plane that was caused by the interlayer stacking of the conjugated aromatic system, whereas the latter peak was attributed to the (100) plane due to an in-plane structural packing motif. These two diffraction peaks are consistent with the results that have been reported in the literature^6, 9, 18, 37, 45, 46^. The diffraction patterns of CNTi composites (Fig. 4b–d) were similar to that of bare TiO_2_ without the distinctive peaks of C_3_N_4_. This is can be ascribed to either (i) the low quantity of C_3_N_4_ in the composites or (ii) the peak overlap that relates to the interlayer stacking of the conjugated aromatic system with the peak at 27.5° for rutile TiO_2_^7, 14, 47^.

**Figure 5:**
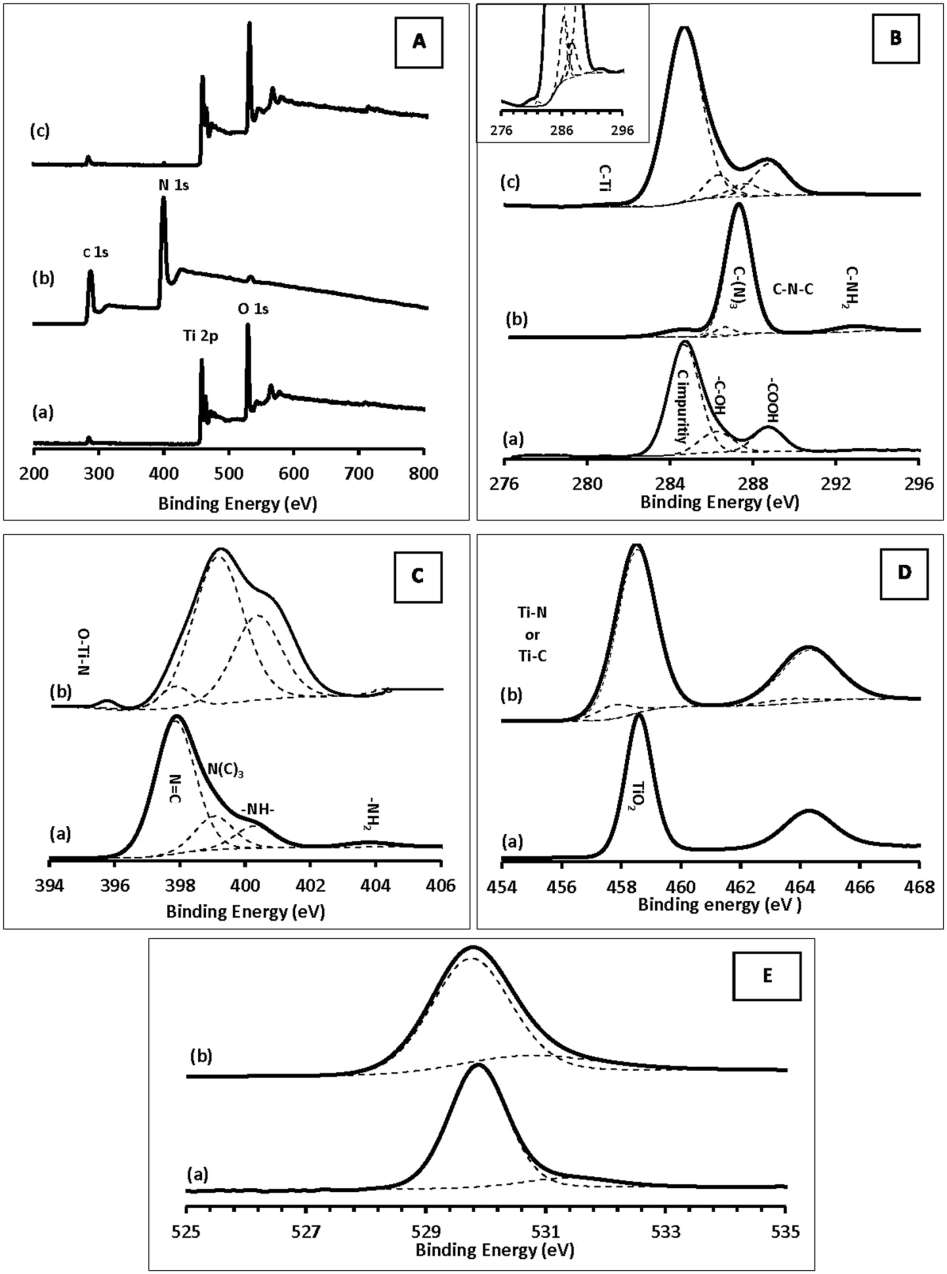
**(A)** XPS survey of (a) TiO_2_ (b) C_3_N_4_ and (c) CNTi, (**B**) C 1 s region of (a) TiO_2_, (b) C_3_N_4_ and (c) CNTi, (**C**) N 1 s region of (a) CN and (b) CNTi, (**D**) Ti 2 P region of (a) TiO_2_ and (b) CNTi, (**E**) O 1 s region of (a) TiO_2_ and (b) CNTi.

XPS measurements were performed to obtain information regarding the surface chemical composition along with their oxidation states. The survey spectra show Ti, C and O peaks in bare TiO_2_ (Fig. 5Aa), C, O and N peaks in C_3_N_4_ (Fig. 5Ab) and C, N, Ti and O peaks in the CNTi composite (Fig. 5Ac). Figure 5B shows the C region of bare TiO_2_, CN and CNTi. The deconvolution of the C region of bare TiO_2_ (Fig. 5Bc) revealed three spectral peaks at 284.7, 286.2 and 288.8 eV, which were ascribed to adventitious carbon, -C-OH and -COOH groups, respectively^22^. In the C_3_N_4_ spectrum (Fig. 5Bb), besides the spectral peak at 284.7 eV, which corresponds to C impurities, three peaks at binding energies of 286.6, 287.4 and 292.8 eV were allocated to the C-N-C, C-(N)_3_ and C-NH_2_ groups, respectively^6^. The CNTi composite preserved all the carbon functional groups that existed in both TiO_2_ and C_3_N_4_, as indicated in the devolution of its C region (Fig. 5Bc). However, a new peak at a lower binding energy of 281.1 eV appeared, which can be assigned to the C-Ti bond^48^. The regional N 1 s spectra for C_3_N_4_ and CNTi composites are presented in Fig. 5C. The spectrum of C_3_N_4_ (Fig. 5Ca) was deconvoluted into four peaks, which are attributed to N = C at 397.9 eV, N-(C)_3_ at 399.1 eV, -NH- at 400.3 eV and -NH_2_ at 403.8 eV^11, 15^. All the N functional groups existed in CNTi and a new peak at a lower binding energy of 395.4 eV, which may be ascribed to an O-Ti-N bond (Fig. 5Cb). Figure 5D displays Ti 2 P spectra of pure TiO_2_ compared with that of the CNTi composite. The Ti 2p_3/2_ and 2p_1/2_ spin-orbit coupling peaks of bare TiO_2_ appeared at 458.7 and 464.3 eV, respectively, with a spacing of 5.6 eV, which was ascribed to Ti^4+^ species in the TiO_2_ cluster. However, this peak showed a broadening with a 0.2 eV shift of Ti 2p_3/2_ to a lower binding energy, whereas the 2p_1/2_ remained un-shifted after loading C_3_N_4_ on TiO_2_. The deconvolution of the Ti region of the CNTi composite (Fig. 5Cd) revealed the existence of Ti-N or/and Ti-C (at 457.8 and 463.3 eV for Ti 2p_3/2_ and 2p_1/2_, respectively) with a spacing of 5.5 eV besides the TiO_2_ main peak. Therefore, it can be concluded that the oxidation state of Ti (IV) did not change after the loading of C_3_N_4_ on TiO_2_, and there is a chemical bond between the Ti and C and/or N of the C_3_N_4_. The negative shift in the Ti peak of CNTi due to electronic interactions between Ti and N atoms and/or C atoms caused an increase in the electron density on Ti. This was also supported by the existence of O-Ti-N in the N 1 s region (Fig. 5Cb) and the peak of C-Ti in the C 1 s region (Fig. 5Bc). The O 1 s spectrum (Fig. 5Ea) of pure TiO_2_ were fit into two peaks at binding energies of 529.87 and 531.42 eV that were assigned to oxygen in TiO_2_ and the surface hydroxyl, respectively^14, 16, 49^. In comparison with pure TiO_2_, a negative shift of the main O 1 s to 529.2 eV was observed for the CNTi composite. Note that this shift of 0.2 eV in O1s is similar to the shift found in Ti 2p_3/2_ for the CNTi composite, which supports the suggestion of the existence of bonds between Ti and C and/or N and are in good agreement with previous reports^6, 14, 15, 33^. The XPS, XRD and FT-IR studies clearly revealed the successful preparation of nanocomposite material with chemically bound interfaces between C_3_N_4_ and TiO_2_ rather than a physical mixture of two separate g-C_3_N_4_ and TiO_2_ phases.

Argon bombardment was used to etch the CNTi nanocomposite to gain information regarding which material on top of the other. From Table 1, it is obvious that CN covered the TiO_2_ surface since the quantity of TiO_2_ increased while the quantity of carbon decreased with etching.

**Table 1:** Argon etching of 1CNTi at different times.

Etching Time. (s)	Etching Depth, (nm)	C,(%)	Ti,(%)
0	0	9.4	26.5
15	1	2.4	29.2
40	3	1.9	30.7
65	5	1.4	31.5
90	7	1.3	31.9
125	10	1.2	32.0
230	20	1.0	33.3

The elemental analysis of as-prepared C_3_N_4_ provided a C to N ratio of 0.53, which is less than the theoretical value of ideal carbon nitride (0.75 in C_3_N_4_). In addition to nitrogen and carbon, the elemental analysis also detected hydrogen. The presence of hydrogen in the form of N-H and NH_2_ groups was established by FT-IR and XPS analyses, which describes the relatively low C to N ratio.

**Figure 6:**
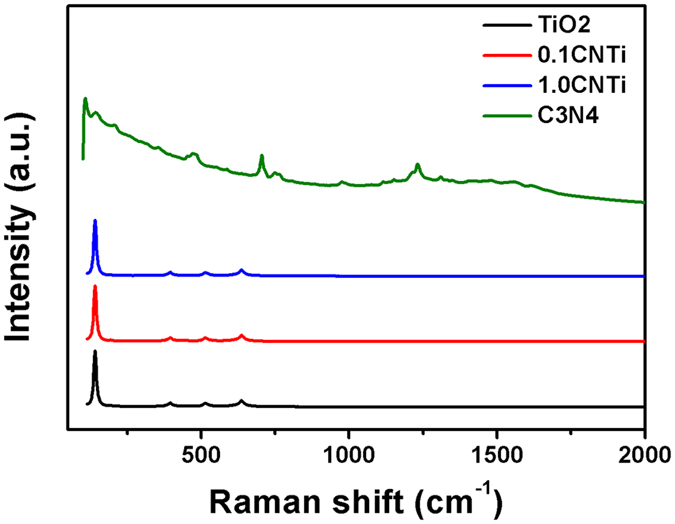
Raman spectra of (**a**) TiO_2_, (**b**) 0.1CNTi, (**c**) 1CNTi, and (**d**) CN.

Figure 6 shows the Raman spectra of TiO_2_, 0.1CNTi, 1CNTi and C_3_N_4_ recorded with a 785 nm laser as the excitation source. The measured Raman spectrum of C_3_N_4_ (Fig. 6d) is similar to the one shown by Tonda *et al*.^50^ in which several characteristic peaks of C_3_N_4_ were revealed with strong peaks at 705 and 1232 cm^−1^ ^51^. Similar to the XRD results, the Raman spectrum of C_3_N_4_ loaded on TiO_2_ did not show any characteristic peaks of C_3_N_4_. Only peaks corresponding to the anatase and rutile phases of TiO_2_ were observed (Fig. 6a–c). This result was also observed by other researchers^51, 52^.

**Figure 7:**
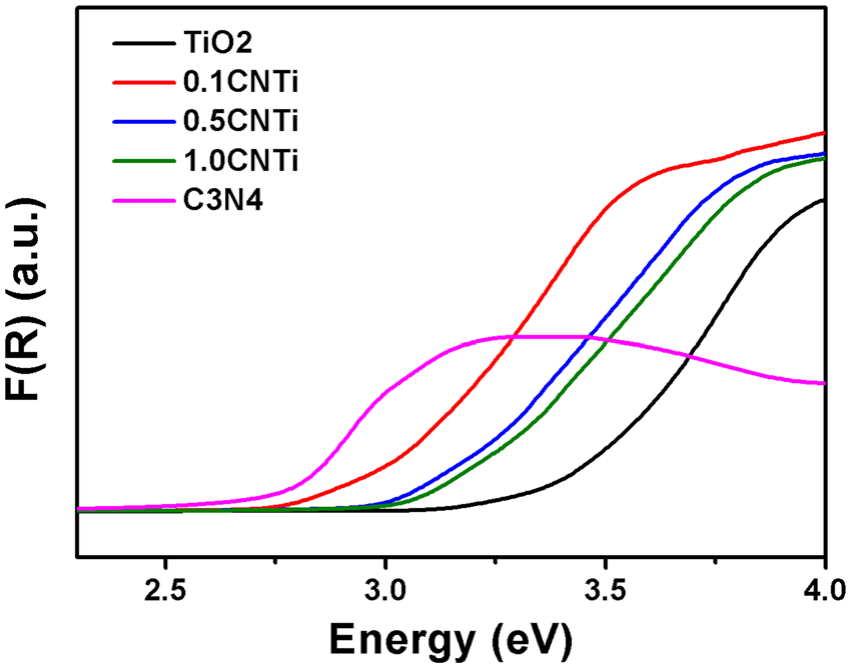
Band gap energies of TiO_2_, 0.1CNTi, 0.5CNTi, 1CNTi and CN (C_3_N_4_) from UV-Vis DRS.

The band gap energies of TiO_2_, 0.1CN, 0.5CN and 1CN were measured and the wavelengths at the absorption edge were calculated and recorded from the UV-Vis diffuse reflectance spectra (Fig. 7 and Table 2). The measurements revealed that the band gap energy of C_3_N_4_ equals 2.75 eV, as reported in the literature^6^. Interestingly, the band gap energy of the CNTi composite increased as the C_3_N_4_ percentage in the composite increased but remained lower than that of the TiO_2_. The composite with the lowest band gap energy (2.95 eV) was 0.1CNTi compared to 3.35 eV for TiO_2_. Therefore, loading CN on TiO_2_ shifted the TiO_2_ absorption wavelength to the visible region and allowed the photocatalytic degradation of phenol using visible light or sunlight irradiation. Note that the BET surface area of 1CNTi composites (49.8–50.6 m^2^ g^−1^) and TiO_2_ (51.1 m^2^ g^−1^) were similar. This proves that the surface area was not the controlling factor for this photocatalytic study.

**Table 2:** Wavelengths at the absorption edge for TiO_2_, 0.1CNTi, 0.5CNTi, 1CNTi and C_3_N_4_ and their corresponding band gap energies.

Catalysts	Wavelength (nm)	Band Gap Energy
TiO_2_	371	3.35
0.1CNTi	422	2.95
0.5CNTi	407	3.05
1CNTi	401	3.10
C_3_N_4_	452	2.75

### Photocatalytic applications

Phenol degradation was studied using a xenon lamp photoexcitation source in the presence and absence of H_2_O_2_ and/or O_3_. The % of phenol degradation was calculated using Equation 1,

1 $${\bf{D}}{\bf{e}}{\bf{g}}{\bf{r}}{\bf{a}}{\bf{d}}{\bf{a}}{\bf{t}}{\bf{i}}{\bf{o}}{\bf{n}}\, \% =[\frac{({{\boldsymbol{C}}}_{0}-{{\boldsymbol{C}}}_{{\bf{t}}})}{{{\boldsymbol{C}}}_{0}}]\,\times \,100$$

where *C*_0_ is the adsorption equilibrium concentration of phenol before irradiation begins. *C*_t_ represents the residual concentration of phenol after specific time *t* of irradiation.

Apparent first order reaction rate law was found to fit the photocatalytic degradation of phenol with a perfect correlation constant (*R* ^2^) close to unity. The rate constant for the degradation, **K**, was obtained from the first-order plot (Equation 2).

2 $${\bf{L}}{\bf{n}}\frac{{{\boldsymbol{C}}}_{{\bf{t}}}}{{{\boldsymbol{C}}}_{0}}=-{\bf{K}}{\boldsymbol{t}}$$

Note that in absence of the photocatalysts (CNTi or TiO_2_ nanocomposites), there was no degradation of phenol, even in presence of C_3_N_4_ without the TiO_2_ support. Furthermore, the C_3_N_4_ did not peel off the TiO_2_ support during and after the photocatalytic experiments, which can be attributed to the chemical bond between C_3_N_4_ and TiO_2_ during the synthesis process.

**Figure 8:**
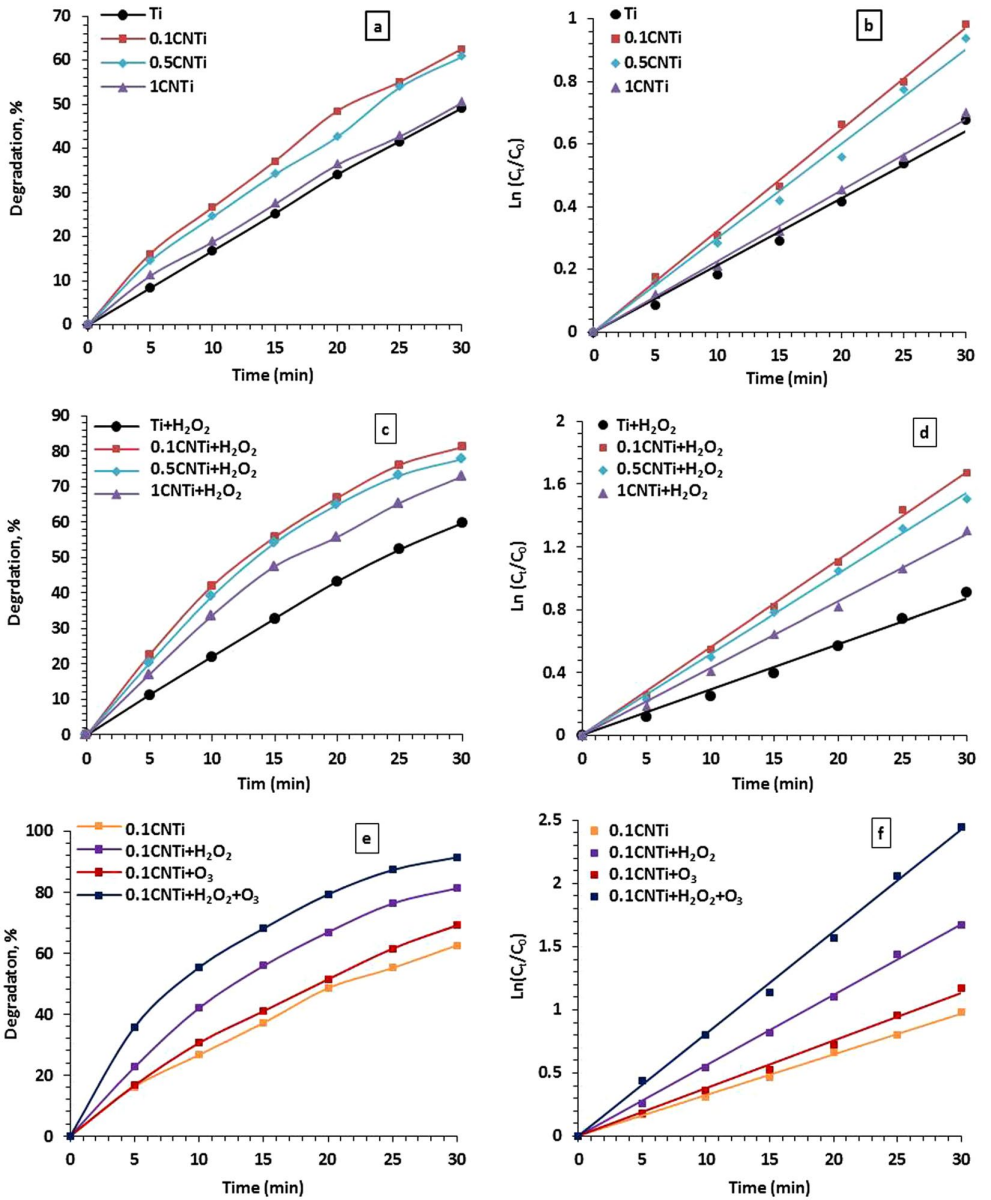
Degradation % (**a**,**c** and **e**) and reaction kinetics (**b**,**d** and **f**) of phenol (20 mgL^−1^) using Ti, 0.1CNTi, 0.5CNTi and 1CNTi nanocomposite photocatalysts under 150 W Xe illumination in the absence (**a** and **b**) and presence (**c** and **d**) of 70 µL of H_2_O_2_ and additional cases using 0.1CNTi in the presence and absence 70 µL of H_2_O_2_ (**e** and **f**) and/or 4 ppm of O_3_ (**e** and **f**).

Figure 8 shows the degradation % (a, c and e) and reaction kinetics (b, d and f) for 20 mgL^−1^ phenol using Ti, 0.1CNTi, 0.5CNTi and 1CNTi nanocomposite photocatalysts under 150 W Xe illumination in the absence (a and b) and presence (c and d) of 70 µL of H_2_O_2_. Additionally, other cases utilizing 0.1CNTi in the presence and absence of 70 µL of H_2_O_2_ (e and f) and/or 4 ppm of O_3_ (e and f) were included. Table 3 summarizes the degradation rate constants in min^−1^ and TOC results. The following conclusions were obtained from Fig. 8 and Table 3. First, in all cases, the photodegradation percentage increased with time regardless of the type of photocatalyst. Second, all CNTi composites showed better photocatalytic activity compared with bare TiO_2_. An increase in the C_3_N_4_ percentage from 0.1 to 1% in the CNTi composites decreased the photocatalytic activity, and 0.1CNTi showed the best performance in terms of the phenol degradation rate. Furthermore, the phenol degradation rate increased with the addition of H_2_O_2_ regardless of the photocatalyst type. This is mostly due to the increase in hydroxyl radical formation and retardation of the electron/hole (e/h^+^) pair recombination^53^ due to the electron acceptor property of the C_3_N_4_ where electrons move from TiO_2_ to the carbonaceous materials inhibiting the recombination between the electrons and the holes.

3 $${{\rm{H}}}_{2}{{\rm{O}}}_{2}+{{\rm{e}}}_{\mathrm{CB}\,}^{-}\to {}^{\cdot }{\rm{O}}{\rm{H}}+{{\rm{OH}}}^{-}$$

4 $${{\rm{H}}}_{2}{{\rm{O}}}_{2}\to {}^{\cdot }{\rm{O}}H\,+{}^{\cdot }{\rm{O}}{\rm{H}}$$

In the case of H_2_O_2_, the addition of O_3_ to the reaction mixture increased the rate of phenol oxidation. However, the rate of phenol degradation in presence of H_2_O_2_ was higher than in the presence of O_3_ regardless of the type of photocatalyst. In addition, the presence of both oxidants (O_3_ + H_2_O_2_) greatly increased the photodegradation rate compared with the case where one oxidant was used. The 0.1CNTi composite demonstrated 91.4% phenol degradation within 30 min with rate of 0.08 min^−1^ when both O_3_ and H_2_O_2_ were used under a Xe illumination of 150 W. Miranda el al. obtained 85% conversion of phenol within 120 min under UV irradiation using g-C_3_N_4_/TiO_2_ composite prepared by impregnation methods^54^. Zhao and co-workers obtained 96.4% phenol degradation within 60 min under a Xe illumination of 500 W (20 ppm phenol and 1 gL^−1^ CNTi)^47^. Zhou *et al*. tested TiO_2_/Co-g-C_3_N_4_ catalyst for phenol degradation. The degradation of 10 ppm phenol reached 100% within 40 min under 300 W xenon illumination^55^. Yao and his group tested TiO_2_-CdS-gCNNSs composite for degradation of 10 ppm phenol under visible light irradiation. They obtained 80% degradation within 300 min^56^. S. Obrego’n and G. Colo’n found that the modification of TiO_2_/g-C_3_N_4_ composite with Pt and Mn improved the photocatalytic activity of phenol degradation under UV radiation. The best phenol rate was obtained 10.6 × 10^−8^ mol/L/s over Pt-TiO_2_/g-C_3_N_4_-MnOx composite^57^.

**Table 3:** Rate constant (min^−1^), rate coefficient, degradation % (%D) and degradation % from TOC using TiO_2_, 0.1CNTi, 0.5CNTi and I CNTi in the presence and absence of H_2_O_2_ and additional cases using 0.1CNTi with O_3_ and both H_2_O_2_ and O_3_.

parameter	Catalyst									
	TiO_2_		0.1% CNTi		0.5CNTi		1CNTi		0.1CNTi + O_3_	0.1CNTi + H_2_O_2_ + O_3_
	−	+H_2_O_2_	−	+H_2_O_2_	−	+H_2_O_2_	−	+H_2_O_2_		
r	0.021	0.030	0.032	0.056	0.030	0.051	0.023	0.043	0.038	0.080
R^2^	0.99	0.99	1.00	1.00	0.99	1.00	1.00	1.00	0.99	0.99
% D	49.1	61.3	62.6	81.2	60.7	77.8	49.7	72.9	69.1	91.4
TOC	54.4	72.8	63.1	81.3	59.9	78.0	49.9	73.0	71.08	92.1

Finally, a comparison of the phenol degradation % based on UV spectrophotometer and TOC measurements indicated similar results when the CNTi nanocomposite photocatalyst was used. By contrast, when TiO_2_ was used, the phenol degradation % from the UV-Vis spectrophotometer measurement was higher than that from the TOC measurement. Therefore, it can be concluded that CNTi nanocomposites undergo complete oxidation in contrast to that of the TiO_2_ surface where intermediates were formed. Su *et al*. reported 65.7% TOC removal of phenol over carbon nitride quantum dots anchored onto TiO_2_ nanotube arrays after 180 min reaction under solar light^58^.

### Stability evaluation

**Figure 9:**
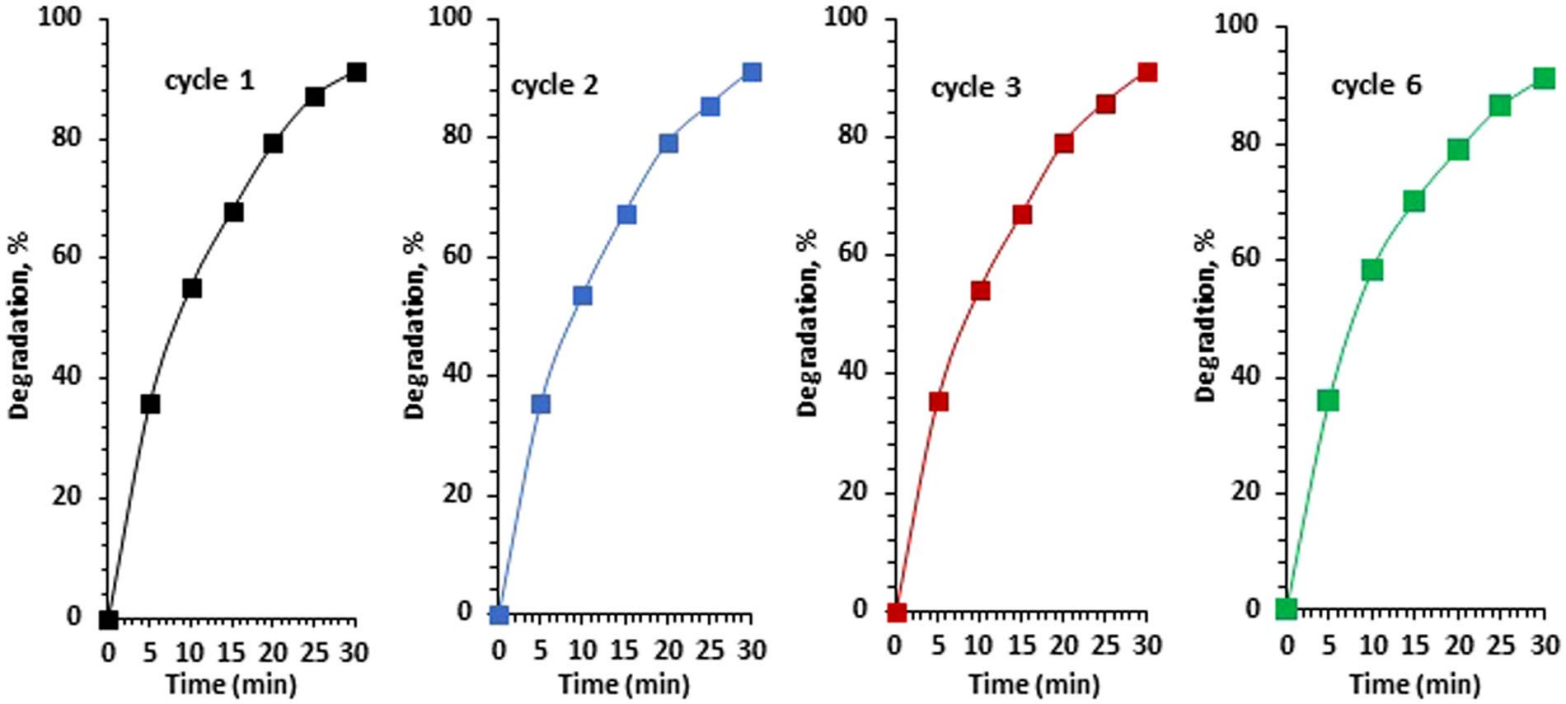
The cycling runs for the degradation of phenol (20 ppm) on 0.1CNTi in presence of H_2_O_2_ and O_3_ under Xe illumination.

Photocatalytic measurements of the 0.1CNTi nanocomposite, which yielded the best degradation rate of phenol (in this study) in the presence of both O_3_ and H_2_O_2_ under Xe illumination, were repeated six times after isolating the photocatalyst. Figure 9 indicates that there was no obvious decline in the photocatalytic activity. In addition, XRD measurements both before and after the reaction was the same (the results are not shown). Therefore, the results demonstrated that the CNTi nanocomposite photocatalyst was stable under the abovementioned experimental conditions.

## Conclusions

Coupling TiO_2_ with C_3_N_4_ (visible light-sensitized semiconductors) has proved to be beneficial for improving the photocatalytic performance due to a synergism that was ascribed to improved light harvesting, enhanced photostability and effective photoexcited charge separation.

The addition of H_2_O_2_ or O_3_ improved the photocatalytic degradation rate of phenol. However, the rate dramatically increased when a trace amount of O_3_ gas (4 ppm) was added to H_2_O_2_ in the reaction mixture in the photocatalytic reactor, which was attributed to the increase in the rate of ozone degradation and, consequently, the rate of hydroxyl radical formation in the presence of H_2_O_2_.

The best C_3_N_4_ ratio in the CNTi composite, which yielded the highest degradation rate of phenol, was 0.1% C_3_N_4_. This photocatalyst was stable under experimental conditions, and its performance was drastically enhanced in the presence of both O_3_ and H_2_O_2_.

Finally, based on the results, the CNTi nanocomposite photocatalyst can be considered to be a promising candidate for the treatment of wastewater contaminated with phenol, especially when H_2_O_2_ with traces of O_3_ gas is used.
